# Transcriptome-wide single nucleotide polymorphisms related to electric organ discharge differentiation among African weakly electric fish species

**DOI:** 10.1371/journal.pone.0240812

**Published:** 2020-10-27

**Authors:** Julia Canitz, Frank Kirschbaum, Ralph Tiedemann

**Affiliations:** 1 Unit of Evolutionary Biology/Systematic Zoology, Institute of Biochemistry and Biology, University of Potsdam, Potsdam, Germany; 2 Department of Crop and Animal Science, Faculty of Life Science, Humboldt-Universität zu Berlin, Berlin, Germany; National Cheng Kung University, TAIWAN

## Abstract

African weakly electric fish of the mormyrid genus *Campylomormyrus* generate pulse-type electric organ discharges (EODs) for orientation and communication. Their pulse durations are species-specific and elongated EODs are a derived trait. So far, differential gene expression among tissue-specific transcriptomes across species with different pulses and point mutations in single ion channel genes indicate a relation of pulse duration and electrocyte geometry/excitability. However, a comprehensive assessment of expressed Single Nucleotide Polymorphisms (SNPs) throughout the entire transcriptome of African weakly electric fish, with the potential to identify further genes influencing EOD duration, is still lacking. This is of particular value, as discharge duration is likely based on multiple cellular mechanisms and various genes. Here we provide the first transcriptome-wide SNP analysis of African weakly electric fish species (genus *Campylomormyrus*) differing by EOD duration to identify candidate genes and cellular mechanisms potentially involved in the determination of an elongated discharge of *C*. *tshokwe*. Non-synonymous substitutions specific to *C*. *tshokwe* were found in 27 candidate genes with inferred positive selection among *Campylomormyrus* species. These candidate genes had mainly functions linked to transcriptional regulation, cell proliferation and cell differentiation. Further, by comparing gene annotations between *C*. *compressirostris* (ancestral short EOD) and *C*. *tshokwe* (derived elongated EOD), we identified 27 GO terms and 2 KEGG pathway categories for which *C*. *tshokwe* significantly more frequently exhibited a species-specific expressed substitution than *C*. *compressirostris*. The results indicate that transcriptional regulation as well cell proliferation and differentiation take part in the determination of elongated pulse durations in *C*. *tshokwe*. Those cellular processes are pivotal for tissue morphogenesis and might determine the shape of electric organs supporting the observed correlation between electrocyte geometry/tissue structure and discharge duration. The inferred expressed SNPs and their functional implications are a valuable resource for future investigations on EOD durations.

## Introduction

**Fig 1:**
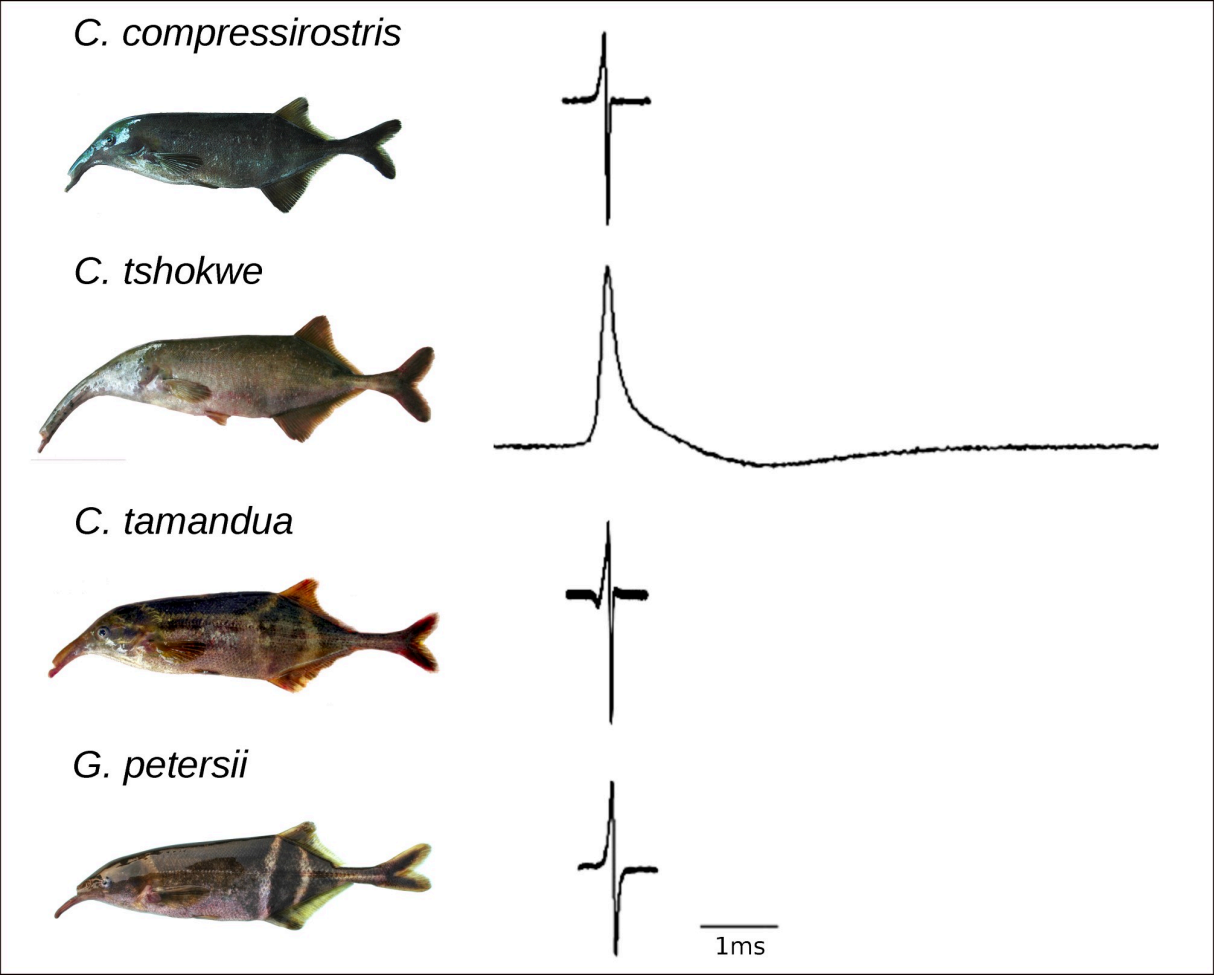
Appearance and EOD waveforms of four mormyrid species used for this study. The morphological shape of each target species is shown, while body sizes may vary. Species-specific EOD wave forms are represented in relation to a 1ms time scale.

In closely related teleost fish, species-specific differences can be observed in morphology, behavior, reproduction, and communication [1–4]. These trait differences are often adaptive, especially if the species have evolved (or at least occur) in sympatry [5–8]. African weakly electric fishes of the family Mormyridae (Osteoglossiformes; Teleostei) comprise numerous sympatric closely related species differing in their electric sense. Mormyrids evolved an electric organ enabling them to produce electric organ discharges (EODs) for orientation and communication, i.e., mate recognition and species discrimination [9–14]. The main structure of electric organs is formed by tens of specialized cells, called electrocytes, which have a disk-like shape and are stacked in cylindrical columns [15–21]. Either the anterior or the posterior face of electrocytes gives rise to several finger-like evaginations fusing in a stalk, serving as the interface to the electromotor neuron. Those stalks can penetrate the electrocyte and occur as single or multiple stalk-systems. The electrocyte faces can be smooth or unevenly invaginated with papillae or folds increasing the membrane’s surface [15, 16]. The membrane is excitable and packed with different ion channels, such that each single electrocyte is independently capable of generating an action potential [16]. The simultaneous release of all action potentials forms the weak pulse-type discharge which varies among closely related species in the number and orientation of phases as well as in its duration. In several African mormyrid weakly electric fish, slight differences in EOD characteristics occur between sexes and populations, while this was never detected in our focus genus *Campylomormyrus*, rendering their EOD a species-specific trait [14, 18]. Currently, 15 *Campylomormyrus* species are morphologically described and for 9 of them the EOD is known. The main morphological differences regard the head shape, and especially the length and curvature of the elongated snout. Among the four mormyrid species used in this study, *Campylomormyrus tshokwe* possesses the longest snout (Fig 1). Waveforms of the EOD also vary among the species. They can be bi- or triphasic with putatively ancestral pulse duration mostly shorter than 400μs (*Campylomormyrus compressirostris*; *Campylomormyrus tamandua; Gnathonemus petersii*). A significantly longer EOD (> 4ms) occurs in *C*. *tshokwe* and is assumed to be the derived character state within the genus *Campylomormyrus* (Fig 1) [22, 23].

In mormyrids, there is evidence that different patterns of electrocyte geometry, like electrocyte penetrations, cause different EOD waveforms. The electric organ of several mormyrid species has been histologically analyzed and a relation between electrocytes being stalk-penetrated or not and the EOD phase number could be observed [15–19]. Further, species with a multiple-stalk system are found to have a longer EOD than those with a single-stalk system [19]. Stalk size as well as the size of the electrocytes also seem to play a role in determining pulse duration. This is observed in *Brienomyrus* species where longer EODs emitted by males coincide with thicker electrocytes and larger stalks, relative to females [24, 25]. A comparable correlation can also be found among differentially discharging species of *Campylomormyrus* [18]. Moreover, a link between the increase of the electrocyte surface at the anterior face and EOD length has been found after hormone treatment (e.g., with testosterone) [24–27]. As these experiments are mainly performed in species with sex-specific EOD differences, it is not clear, whether hormones also contribute to inter-specific variation.

Further crucial components for an EOD generation are different types of ion channels integrated in the electrocyte membrane. They are responsible for generating the action potential, as they regulate the in- and out-flux of sodium and potassium ions. Hence, variations in the abundance or molecular structure of ion channels may also contribute to different pulse durations. All these components are encoded in the genome. Sequence variation of genes coding for major cellular components like the ion channels has been assessed in relation to EOD duration in several mormyrid species [28–31], but could not fully explain the observed variation in EOD characteristics.

Mutations can affect a particular phenotypic trait in different ways. Single point mutations in the coding region of a particular gene can change the protein sequence and hence, its function, resulting in variation of a phenotypic trait [32–34]. Often, however, traits have a quantitative inheritance, i.e., they depend on several or many genes and multiple mutations in these genes may contribute to trait variability. Regarding the ion channels, single nucleotide polymorphisms (SNPs) have been detected between wave-type and pulse-type African weakly electric fish in one of the two paralogous genes of the voltage-gated potassium ion channel *kcna7a* [35]. Among *Campylomormyrus* species with shorter and longer EODs, SNPs are also found in one paralog of the voltage-gated sodium channel gene (*scn4aa*) [31]. In those genes, non-synonymous SNPs likely alter the protein function and thus, can contribute to a change of the EOD duration. Mutations can also affect gene expression by impairing promoter and transcription factor interactions, modifying the function of regulatory proteins like activators/repressors, or changing mRNA conformations (stability) [36–38]. Indeed, the elongated EOD in *C*. *tshokwe* is associated with significantly elevated expression in several ion channel genes [30] but the regulatory elements behind this up-regulation are not yet known. Moreover, studies of other weakly electric fish used differential gene expression to investigate candidate genes and their impacts on the electric activity [39–42]. EOD characteristics are likely encoded by multiple genes being part of various cellular processes. Thus, an identification of genes and processes related to the EOD in weakly electric fish will provide further insights into its evolution and the mechanisms which cause the different pulse duration.

In this study we aim at identifying further genes potentially influencing the pulse duration by using RNA sequencing data of the electric organ of three *Campylomormyrus* and one *Gnathonemus* species with different EOD durations (Fig 1) [40]. Specifically, we look for putative candidate genes with non-synonymous SNPs which are related to EOD duration. Such expressed single mutations may have an impact either on gene expression or protein functions. Furthermore, we put our data in the context of gene ontology to infer those biological processes which are likely involved in the determination of EOD duration. As EOD differentiation is likely due to divergent selection among closely related species, genes with species-specific mutations may be more common for those biological processes and mechanisms which are relevant for a derived elongation of pulse duration. Conceptually, we screen two closely related species with short and long EOD, respectively, for species-specific non-synonymous SNPs, having two further species with ancestral EOD characteristics as outgroup to discern putatively derived from ancestral SNP states.

## Results

### Transcriptome assemblies

In total, 66986804, 117994270, 41018678 and 99097520 high quality reads were used to assemble de novo the transcriptomes of *C*. *compressirostris*, *C*. *tshokwe*, *C*. *tamandua* and *G*. *petersii*, respectively. The four Trinity assemblies contained between 141384 and 218372 contigs with a N50 length ranging from 1393 to 1881 bp (Table 1). According to the BUSCO analysis, the four transcriptomes matched a proportion of 62.5% to 76.9% of the Actinopterygii core genes data set, depending on the species (Table 1). After isolating transcripts with the longest open reading frame (LORF) by following the TransDecoder pipeline, transcriptome size decreased by around 70%, leaving a remainder of 50241 transcripts for *C*. *compressirostris*, 65330 for *C*. *tshokwe*, 48929 for *C*. *tamandua*, and 52226 for *G*. *petersii* (Table 1). The identification of orthologous sequences among the four transcriptomes outputted a total number of 36285 orthogroups containing 16661 orthogroups present in all species of which 5284 were Single Copy Orthogroups (SCO). Subsequent alignment and filtering steps reduced the data set to a final number of 5071 SCO.

**Table 1:** Assembly statistics of the four transcriptomes.

	*C*. *compressirostris*	*C*. *tshokwe*	*C*. *tamandua*	*G*. *petersii*
Number of processed reads	66986804	117994270	41018678	99097520
Number of contigs	160665	218372	141384	176155
N50	1393	1873	1881	1643
Number of transcripts with LORF	50241	65330	48929	52226
BUSCO completeness (Actinopterygii core gene set)	62.5%	76.9%	64.4%	68.0%

### Approach I: Identification of candidate genes potentially related to EOD elongation in *C*. *tshokwe*

The goal of approach I was the identification of distinct putative candidate genes supposed to play a role in the EOD elongation in *C*. *tshokwe*. Candidate genes were identified by fulfilling two conditions: an inferred positive selection among the four mormyrid species (ω > 1) and the occurrence of species-specific non-synonymous SNPs in *C*. *tshokwe*. The PAML/codeml analysis for inferring positive selection revealed 131 SCOs with ω > 1. Among these SCOs, 39 sequences had at least one *C*. *tshokwe*-specific non-synonymous SNP. For 27 of them, only *C*. *tshokwe* was deviant, while *C*. *compressirostris* was identical to the outgroup taxa. These genes were considered as potential candidates related to the elongated EOD in *C*. *tshokwe* (see Materials and Methods for details). Twenty of these candidate genes had 1, six had 2 and one had 3 *C*. *tshokwe*-specific non-synonymous substitutions (Table 2).

**Table 2:** List of 27 candidate genes potentially related to the EOD elongation of *C*. *tshokwe*.

Gene	Gene name	SNP position **	Amino acid change	Paml/codeml ω value	General function
*trs2*	TSR2, Ribosome Maturation Factor	A 432 G	K 138 E	1.058	• transcriptional regulation
					• apoptosis
*znf32*	Zinc Finger Protein 32	T 110 C	V 37 A	3.352	• transcriptional regulation
		T 452 C	V 151 A		
*mapkbp1*	Mitogen-Activated Protein Kinase Binding Protein 1-like	G 122 A	S 40 N	1.689	• immune system response
					• regulatory function
*cd40*	Tumor Necrosis Factor (TNF) Receptor superfamily member 5	T 330 G	D 110 E	1.295	• immune system response
*sprtn*	SprT-like N-Terminal Domain (Spartan)	T 919 C	S 307 P *	1.215	• DNA damage response
*trim56*	Tripartite Motif Containing 56	G 271 A	V 91 I	1.874	• immune system response
		G 413 A	G 138 E		
*nfu1*	NFU1 iron-sulfur cluster scaffold homolog	A 454 G	I 152 V	2.144	• iron-sulfur cluster biogenesis
*wdyhv1*	WDYHV Motif Containing 1	G 305 C	R 102 P	1.613	• cellular protein modification process
*cir1*	Corepressor Interacting With RBPJ, 1	T 1000 C	F 334 L	2.714	• transcriptional regulation
		A 1238 G	D 413 G		• signal transduction
*dnaja1*	DnaJ Heat Shock Protein Family (Hsp40) Member A1	T 838 A	S 280 T	4.105	• protein folding
		T 1034 C	V345 A		• regulation of androgene receptor activity
*cstl1*	Cystatin-like	G 92 A	G 31 E	1.769	• regulatory function
*ewsr1*	EWS RNA-binding protein 1	A 598 G	T 200 A*	2.042	• neuron development
					• transcriptional regulation
*anxa3b*	Annexin A3b	A 544 G	N 182 D/E	1.092	• cell morphogenesis
					• membrane permeability
*rev3I*	REV3 Like, DNA Directed Polymerase Zeta Catalytic Subunit	A 400 G	T 134 A*	2.601	• DNA repair
					• cell proliferation
*ap4b1*	Adaptor Related Protein Complex 4 Subunit Beta 1	G 404 A	G 135 D	1.242	• localization
		C 806 T	A 269 V		
*vcam1*	Vascular Cell Adhesion Molecule 1	G 77 A	A 26 N	1.090	• cell-cell recognition
*znfx1*	Zinc finger, NFX1-type containing 1	T 173 C	V 58 A	1.776	• DNA-binding
					• transcription factor activity
*hbba1*	Hemoglobin, beta adult 1	T 57 G	F 19 L	2.190	• oxygen transport
*Scimp*	SLP Adaptor and CSK Interacting Membrane Protein	T 256 C	S 87 P	1.716	• immune synapse formation
					• signal transduction
*znf678*	Zinc Finger Protein 678	T 491 C	V 164 T/A	1.120	• transcriptional regulation
*igdcc4*	Immunoglobulin superfamily DCC subclass member 4	A 428 G	N 143 S	1.553	• binding
*atp5mf*	ATP Synthase Membrane Subunit F	A 275 G	D 92 G/S	1.294	• ATP production
*Rgmb*	Repulsive Guidance Molecule BMP co-receptor b (3'UTR)	G 8 C	W 3 S	1.453	• development of nervous system
*cunh2orf42*	Chromosome unknown C2orf42 homolog	A 52 G	K 18 E	1.522	• integral component of membrane
		T 97 C	S 33 P		
*-*	uncharacterized LOC111853234 transcript variant X2	G 209 A	G 70 E	1.595	-
*-*	uncharacterized protein LOC109871595	A 268 G	T 90 A	1.248	-
-	unknown gene	T 65 C	V 22 A	1.801	-
		C 150 G	D 50 E		
		T 166 C	W 56 R		

* Amino acid substitutions predicted to impair/alter protein function.

** SNP position refers to the SCO alignments.

The BLASTn analysis assigned 24 sequences to known coding genes, 2 sequences to uncharacterized proteins/regions and one sequence had no blast hit. Sixteen genes could be assigned to GO terms including mainly the terms 'binding' (75%) in the category Molecular Function, 'cellular process' (25%), 'metabolic process' (25%) as well as 'response to stimuli' (20%) in the category Biological Process, and 'cell'/'cell part' (50%) and 'membrane' (15%) in the category Cellular Component (S1 Fig). The KEGG pathway annotation yielded 7 genes involved in 27 pathways, mainly belonging to the categories 'Human Diseases' (5 genes in 15 pathways), 'Environmental Information Processing' (3 genes in 5 pathways) and 'Organismal System' (3 genes in 4 pathways) (S1 Table). In three candidate genes, *C*. *tshokwe*-specific SNPs led to amino acid substitutions that likely impair/alter the protein function according to the MAPP analysis: SprT-like N-Terminal Domain (*sprtn)*, EWS RNA binding protein 1a *(ewsr1a)*, and REV3 Like, DNA Directed Polymerase Zeta Catalytic Subunit *(rev3l*) (Table 2).

### Approach II: Comparative annotation analyses of orthogroups between *C*. *compressirostri*s and *C*. *tshokwe*

Approach II was established to identify those biological mechanisms which are likely to take part in the EOD elongation of *C*. *tshokwe*. By SNP calling of multiple sequence alignments, 2203 and 1195 SCOs with species-specific SNPs were found for *C*. *tshokwe* and *C*. *compressirostris*, respectively. At least one non-synonymous SNP was detected in 824 SCOs for *C*. *tshokwe* and 825 for *C*. *compressirostris*. In 261 cases, the same SCO was affected (albeit at different non-synonymous sites), yielding 563 and 564 unique SCOs for *C*. *tshokwe* and *C*. *compressirostris*, respectively. After filtering for SCOs with inferred positive selection (ω > 1), the data set of *C*. *tshokwe* contained 201 SCOs and that of *C*. *compressirostris* 192 SCOs. All the sequences of these SCOs matched the criteria of inferred positive selection and of carrying at least one non-synonymous SNP in only one of the two respective species (*C*. *tshokwe* or *C*. *compressirostris*) and named hereafter "candidate SCO-sequences".

**Fig 2:**
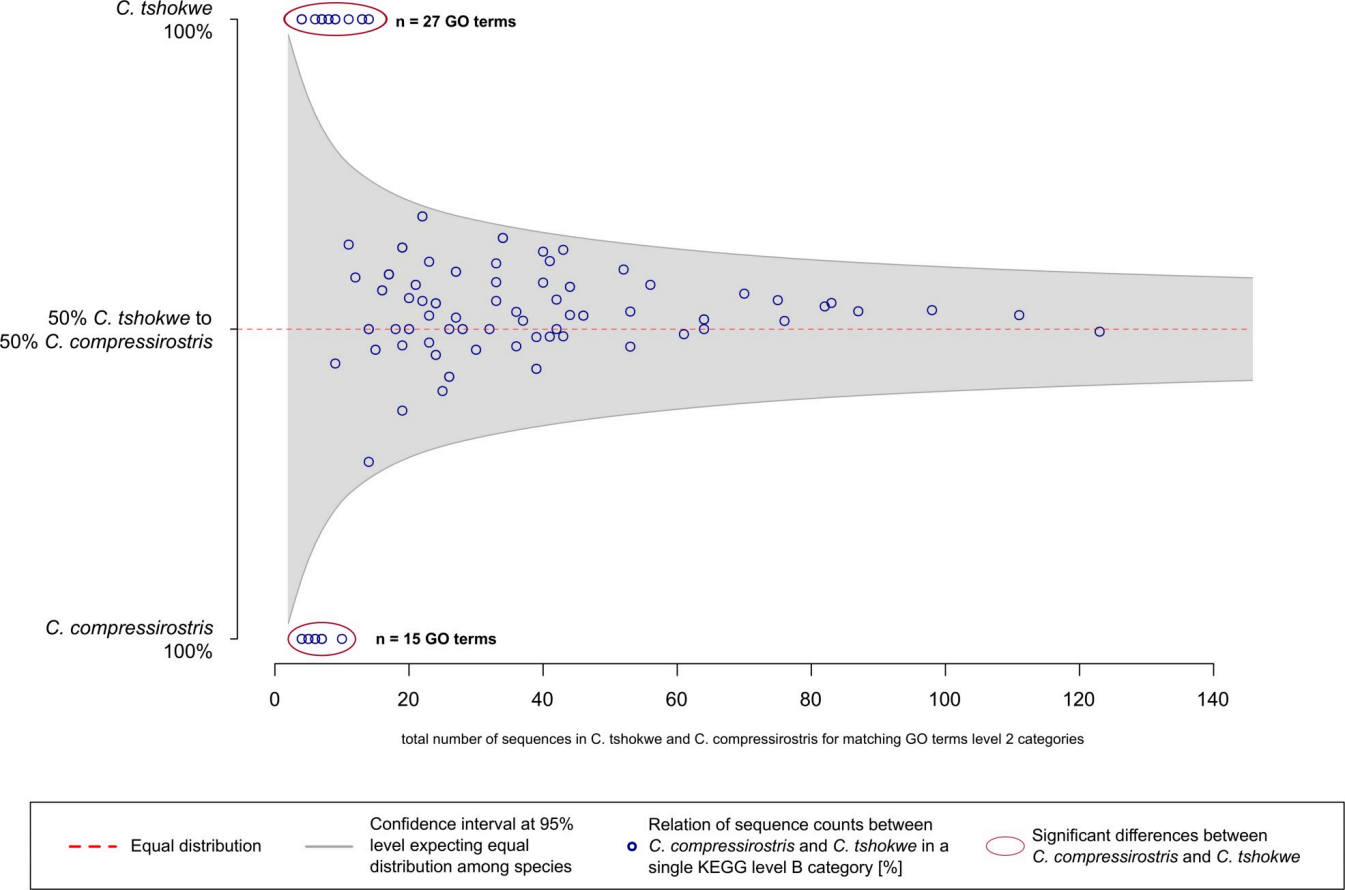
Proportional ratio of candidate SCO-sequences between *C*. *tshokwe* and *C*. *compressirostris* for any annotated GO-term. The figure illustrates the proportional ratio of candidate SCO-sequences between the two *Campylomormyrus* species (y-axis) relative to the total candidate SCO-sequence number for any annotated GO term (x-axis). The 95% confidence interval for an equal ratio (50:50) is depicted as the gray shaded area, rendering dots (i.e., GO terms) outside of the area significant (red circles).

For the identification of biological processes being relevant for the EOD duration, (i) a GO term annotation comparison and (ii) KEGG orthology annotation comparison of level A and B categories were performed among the two species. Regarding the first comparison, Blast2GO assigned 126 GO terms to 110 candidate SCO-sequences of *C*. *tshokwe*, while 116 candidate SCO-sequences were annotated to 114 GO terms for *C*. *compressirostris*. A combination of both annotations resulted in a total of 141 GO terms (S2 Table). For 42 GO-terms, there was a significant difference among the two species in the proportion of candidate SCO-sequences assigned to them. For 27 GO terms, this proportion was significantly higher in *C*. *tshokwe* than in *C*. *compressirostris* (Fig 2). These GO terms were mainly related to molecule binding, ion transport, signal transduction, cell communication, peptides, membrane, extracellular matrix and transcription factor complexes. Conversely, the proportion of candidate SCO-sequences was significantly higher for 15 GO terms in *C*. *compressirostris* (Fig 2). These GO terms were associated with metal ion binding, cytoskeleton and phosphate related metabolic processes as well as cell organelle lumen and cytoskeleton components (S2 Table).

The KEGG orthology analysis (KO) assigned 134 (*C*. *tshokwe*) and 130 *(C*. *compressirostris*) candidate SCO-sequences, respectively, to 145 and 188 KEGG pathways, of which 82 were found in both species, 63 only in *C*. *tshokwe* and 106 only in *C*. *compressirostris* (S3 Table).

For the KEGG level A categories, total candidate SCO-sequence counts were significantly different by the Fisher’s Exact Test among the two species for 3 KEGG level A categories (p < 0.05; Table 3). Candidate SCO-sequences were fewer in *C*. *tshokwe* for the categories 'Environmental Information Processing' (22 vs. 40 candidate SCO-sequences; p = 0.034), ‘Cellular Processes' (25 vs. 45 candidate SCO-sequences; p = 0.031) and ‘Organismal System’ (26 vs. 73 candidate SCO-sequences; p < 0.001). Furthermore, we compared the distribution of candidate SCO-sequences in each KEGG level A category based on the next lower hierarchical KEGG level B and used the Chi^2^ Test to test for significance (p < 0.05; see Materials and Methods for details). The distribution of SCO-sequences to KEGG level B categories differed significantly among the two species within the level A categories ‘Genetic Information Processing’ (p = 0.009), ‘Cellular Process’ (p < 0.001), ‘Organismal Systems’ (p = 0.007), and ‘Human Diseases’ (p = 0.008) (Table 3 and S3 Table).

**Table 3:** Comparison of proportional assignment of candidate SCO data among *C*. *tshokwe* and *C*. *compressirostris* for KEGG level A categories.

KEGG level A category	Fisher Exact Test ^1^	Chi^2^ Test ^2^
	(p-value)	(p-value)
Metabolism	0.691	0.090**
Genetic Information Processing	0.877	0.009**
Environmental Information Processing	0.034*	0.578
Cellular Processes	0.031*	< 0.001**
Organismal Systems	< 0.001**	0.007**
Human Diseases	0.691	0.008**

^1^ Species-wise comparison of the total number of candidate SCO-sequences assigned to the respective level A category.

^2^ Species-wise comparison of candidate SCO-sequence distributions in a KEGG level A category according to the KEGG level B assignment.

* significant (p < 0.05)

** highly significant (p < 0.01).

**Fig 3:**
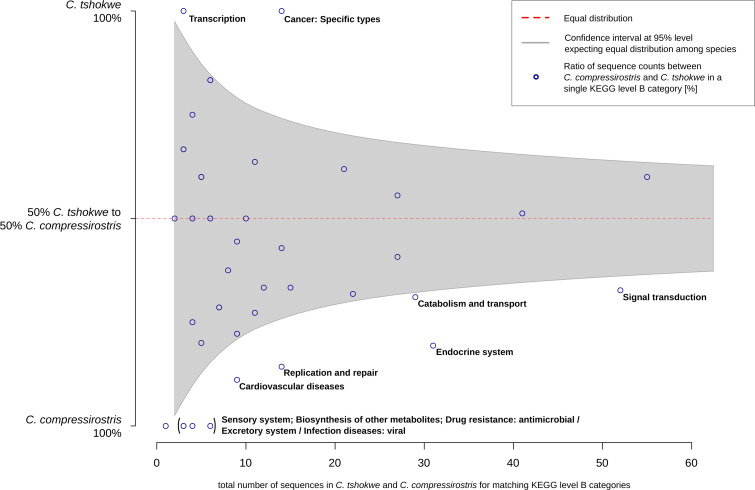
Proportional ratio of candidate SCO-sequences among *C*. *tshokwe* and *C*. *compressirostris* with annotated KEGG pathways. The figure represents the proportional ratio of candidate SCO-sequence counts among both species in a found KEGG level B category (y-axis), relative on the total number of candidate SCO-sequences in the respective category (x-axis). The 95% confidence interval for 50:50 ratio is depicted as gray shaded area, rendering dots (i.e., KEGG level B categories) outside of the area significant.

For a comparative analysis of the next lower hierarchal level (KEGG level B categories), the 251 pathways (82 shared, 63 and 106 unique pathways) were pooled to 45 KEGG level B categories (S3 Table). Among these, 11 categories exhibited significant differences in the percentage of candidate SCO-sequences among species (Fig 3). In *C*. *tshokwe* the 2 categories 'Transcription' and 'Cancer: specific types' were significantly overrepresented in terms of its proportion of candidate SCO-sequences, while in *C*. *compressirostris* 9 KEGG level B categories were overrepresented ('Signal transduction', 'Catabolism and transport', 'Endocrine system', 'Replication and repair', 'Cardiovascular diseases', 'Sensory system', 'Excretory system', 'Biosynthesis of other metabolites' and 'Drug resistance: antimicrobial') (Fig 3).

**Fig 4:**
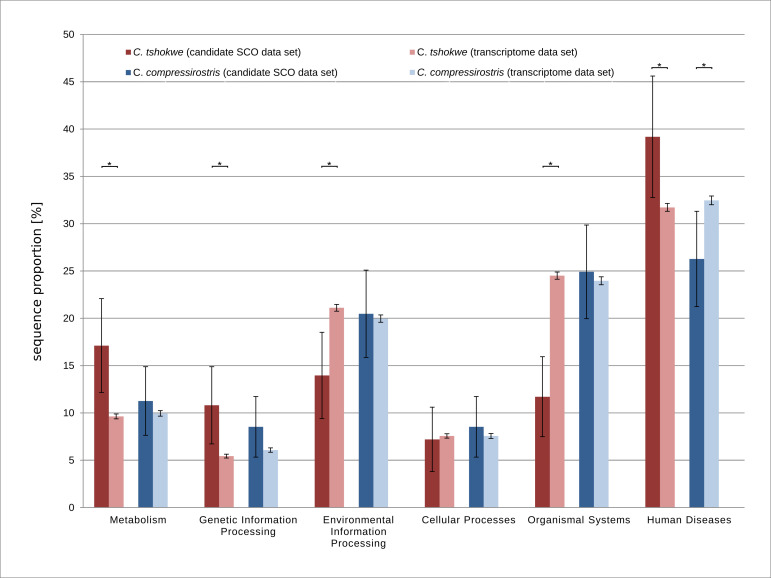
KEGG level A category assignments among the candidate SCO sequences, compared to the entire transcriptome. The bar chart shows the sequence percentage (y-axis) with an annotated KEGG Level A category (x-axis) in the SCO data set and entire transcriptomes of *C*. *tshokwe* (red) and *C*. *compressirostris* (blue). The error bars indicate the 95% confidence interval, taking the total absolute number of candidate SCO-sequences into account (confidence limits of proportions). Asterisks (*) depict significance at p<0.05.

We also compared the annotations of the candidate SCO data set to those of the corresponding transcriptome (KEGG level A categories and GO terms) to evaluate whether certain annotation terms/categories are enriched among candidate SCO-sequences compared to all transcripts. In both species, no GO term was enriched at a FDR threshold of 0.05. Regarding the KEGG level A categories, a significant difference between the candidate SCO and transcriptome data set of *C*. *tshokwe* was observed in 5 out of 6 categories (Fig 4). Here, the categories 'Metabolism', 'Genetic Information Processing' and 'Human Diseases' exhibited a significantly higher and 'Environmental Information Processing' and 'Organismal System' a significantly lower proportion in the candidate SCO data set. In *C*. *compressirostris*, only a single KEGG level A category, 'Human Diseases', yielded a significantly different (i.e., lower) proportion of sequence annotations in the candidate SCO data set compared to the entire transcriptome (Fig 4, S4 Table).

## Discussion

Within mormyrid species, several mechanisms are discussed to explain differences in EOD characteristics, i.e., electrocyte and stalk geometry, variation in hormone levels, and cell membrane excitability [18, 19, 43]. In this study, we identified 27 genes, which (1) are expressed in the electric organ, (2) are inferred to experience positive selection during the evolution of *Campylomormyrus*, and (3) contain at least one non-synonymous mutation in the target species with the derived elongated EOD, i.e., *C*. *tshokwe*. These genes contain expressed genetic variation associated with EOD length differences and can hence be considered as potential candidates underlying this trait.

One of the most abundant functions among the candidate genes is transcriptional regulation. Ten of these genes regulate gene expression directly (e.g., zinc finger) or affect transcription factors and their pathways (e.g., Nuclear Factor kappa B (NFкB) and the Extracellular Signal-Regulated Kinase 1/2 (ERK1/ERK2) signaling cascade). The SLP Adaptor and CSK interacting membrane protein (*scimp*) positively regulates the ERK1/2 pathway which in turn modifies the activity of transcription factors, and hence changes gene expression levels of target genes that are important for the cell cycle progression and cell fate [44]. Additional three genes (*tsr2*, *mapkbp1* and *cd40*) play a role in the regulation of NFкB signaling pathway [45–47]. The protein complex NfкB is a transcription factor regulating the expression of several target genes being responsible for cell proliferation and differentiation as well as apoptosis. Neuroscientific studies proposed that the NFкB pathway mediates long-term changes in synaptic structures and neuronal plasticity via gene expression regulation [48, 49]. The co-repressor interacting with RBPJ1 (*cir1*) negatively regulates the NOTCH signaling pathway which is essential in cell-cell communication and cell differentiation processes at embryonic and adult stages [50–52]. It also plays a role in neuronal function and development, angiogenesis and cardiac valve homeostasis [53–56]. Furthermore, three genes code for zinc finger proteins (*znf32*, *znfx1* and *znf678*). This class of nucleic acid binding proteins has a zinc finger domain interacting with DNA and thus, acts as transcription factor. While the target genes of two of the zinc finger proteins (*znfx1* and *znf678*) are unknown in zebrafish, a knock-down of *znf32* suppresses the *SOX2* transcription which in turn enhances the regeneration of the nervous lateral line system [57]. In other model organisms, these three zinc finger proteins are associated with cancer pathways and epigenetic methylation [58–61]. Transcriptional regulation is also linked to the gene coding for Annexin A3 (*anxa3b*) and the EWS RNA binding protein 1a (*ewsr1a*) [60, 61]. The relevance of this mechanism is corroborated by the GO terms 'cell communication', 'signal transduction' and 'transcription factor complexes' being significantly overrepresented among candidate SCO-sequences in *C*. *tshokwe* compared to *C*. *compressirostris* (S2 Table). Moreover, the KEGG orthology comparison of *C*. *tshokwe* yields the category ‘Genetic Information Processing’ (including processes of transcriptional regulation) to be significantly overrepresented among candidate SCO-sequences, compared to its occurrence in the entire transcriptome (Fig 4). Furthermore, the KEGG level B category ‘transcription’ is more abundant among candidate SCO-sequences of *C*. *tshokwe* than those of *C*. *compressirostris* (Fig 3). These categories refer to RNA polymerase, basal transcription factors and spliceosome. Our results reveal that genes related to processes and components of transcriptional regulation, exhibit an accelerated evolution in *C*. *tshokwe* with species-specific non-synonymous substitutions and inferred positive (divergent) selection. This emphasizes the importance of gene expression regulation for differences in EOD length among species. Indeed, previous studies already showed differential gene expression among *C*. *tshokwe* and *C*. *compressirostris* [30, 40], indicating the impact of transcriptional regulation on EOD waveforms. Future studies should focus on the functional relationship between sequence variation in transcriptional elements revealed in this study and the downstream differential expression in EO-related genes [30, 40]. Transcriptional regulation would provide also a feasible explanation for the remarkable changes in EOD waveforms during the ontogeny of a *Campylomormyrus* fish [62], as expression levels may change during ontogeny [63, 64]. Experimental evidence on ontogenetic expression levels is however still lacking for our target taxa.

Our regulatory candidate genes are mainly associated with cell proliferation, cell differentiation and apoptosis, which holds true also for some non-regulatory candidate genes like the Vascular Cell Adhesion Molecule 1 (*vcam1*), DNA heat shock protein family member A1 (*dnaja1*) and REV3-like DNA directed polymerase subunit zeta (*rev3l*) (Table 2). The functions of their encoded proteins are related to cell expansion, cell survival and cell fate [65–68]. Furthermore, besides its regulatory function, Annexin A3 is mainly responsible for blood vessel and vascular cords formation [69, 70]. According to the MAPP results, the observed species-specific substitutions in the genes *ewsr1a* and *rev3l* are likely to alter the protein function. The function of EWS RNA binding protein 1 (*ewsr1*) is shown to maintain mitotic integrity and proneural cell survival in early developmental stages of zebrafish. A knock-down of this gene results in abnormalities of mitotic spindles, followed by apoptosis and leading to a reduction of the proneural cell number and disorganization of neuronal networks during the early development stages [66]. Knock-down experiments of the second gene, *rev3l*, yield disorganized tissue with significantly reduced cell density [67]. A substitution at a functionally important site in these genes might lead to a functional loss or neofunctionalization and thus, to a modification of subsequent processes affecting cell fate. The association of cell proliferation processes to EOD duration is not only supported by many candidate genes (Approach I), but also by the KEGG categories ‘Human Diseases’ and ‘Cancer: Specific types’ which are significantly more abundant among candidate SCO-sequences of *C*. *tshokwe*. Indeed, most of the cancer pathways are linked to cell proliferation, cell differentiation and apoptosis, all crucial processes of tissue morphogenesis. Expressed genetic variation in these candidate genes may hence contribute to variation in electric organ tissue structures (e.g., multi- or single stalk systems) or cell morphs, supporting the hypothesis of an association between EOD duration and cell geometry [19, 24, 25].

Due to the teleost-specific whole genome duplication 350 mya [71], paralogous copies of essentially all genes emerged, and many of them were retained during teleost evolution. Some of the paralogs may have retained their ancestral function or deteriorated into pseudogenes, but others underwent a neofunctionalization. One of our candidate genes, *anxa3*, is known to have two paralogs in zebrafish (*anxa3a*/ *anxa3b*) [72]. In *C*. *tshokwe* only one gene copy, *anax3b*, was found to be expressed in the electric organ. Annexin A3 has a similar function as some voltage gated ion channels shown to exhibit an electric organ-specific expression [30], i.e., the increase of membrane permeabilization activity and the influx regulation of calcium ions (Ca^2+^) [73] which points out the known importance of ion activity and related proteins during EOD generation.

## Conclusion

To our best knowledge, this is the first transcriptome-wide SNP analysis among African mormyrid weakly electric fish (genus *Campylomormyrus*). Our inferred 27 candidate genes and two molecular biological domains (transcriptional regulation and cell proliferation/cell fate) putatively support a link between tissue structures and EOD durations and provide new opportunities for molecular research regarding the EOD divergence in *Campylomormyrus* and other mormyrids. Genes affecting transcriptional regulation, and subsequent cell proliferation, cell differentiation and apoptosis seem likely to play a crucial role in determining pulse durations. Such processes are important for tissue morphogenesis and cell structures. They have hence the potential to contribute to different electric organ or electrocyte forms. Thus, our results are congruent with the hypothesis of the electric organ geometry not only to affect the shape of EOD pulses, but also their duration.

Biochemical or physiological experiments via, e.g., knock-out trials were out of the scope of this study. A complementary approach could be a co-segregation analysis among EOD phenotypes and genetic variants among F2-species hybrids. Indeed, hybridization of our species is possible [74] and some F1 hybrids are fertile [75], but so far no F2 hybrids could be brought to a stage where they exhibit an adult EOD. Consequently, we can so far not proof a direct link between the inferred candidate genes and processes and the actual electric organ/electrocyte features. Thus, our study provides hypotheses about genes and processes relevant for EOD duration, which future research could build upon.

## Materials and methods

### Transcriptome assemblies

**Fig 5:**
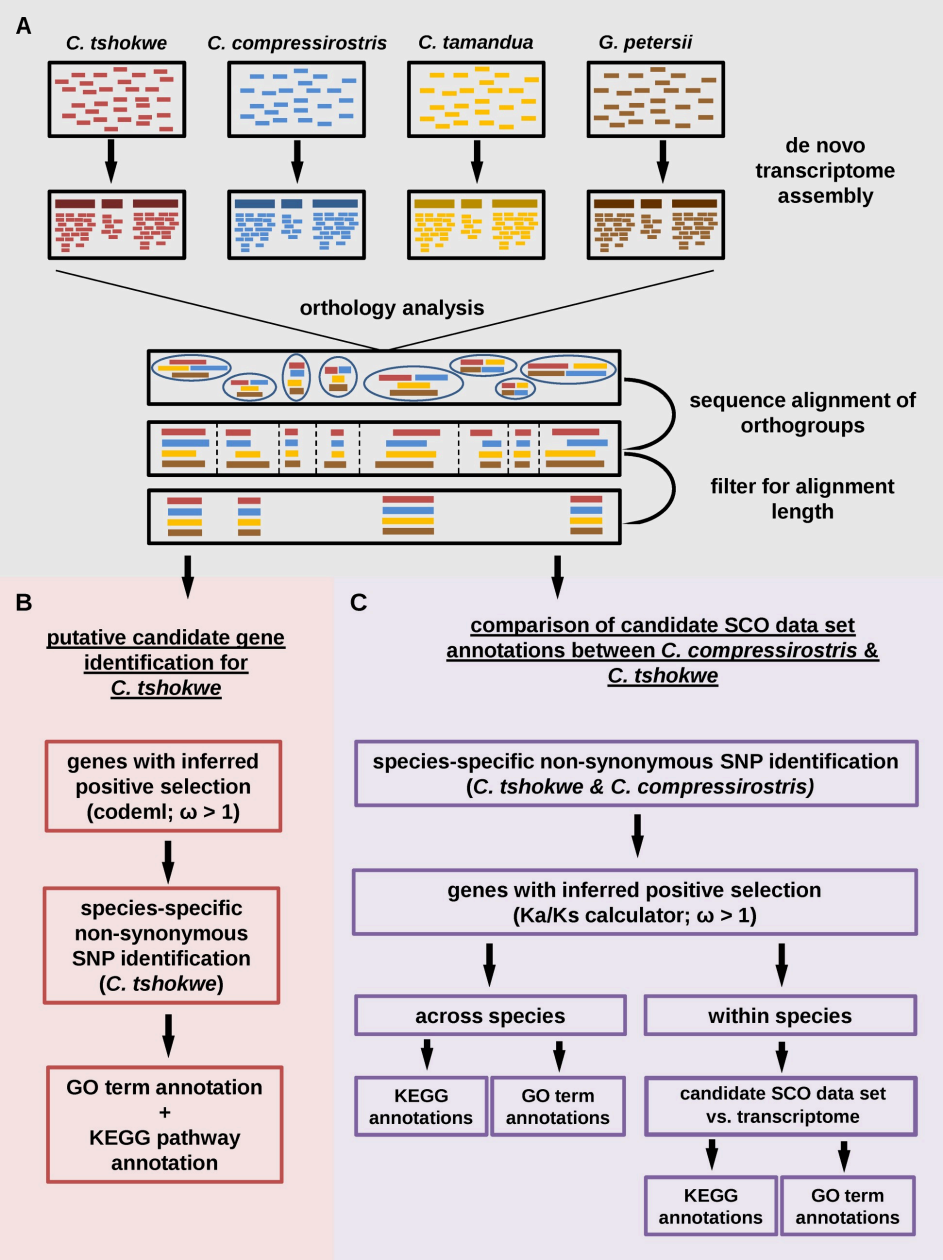
Overview of the workflow of the applied data-analytical approaches. Shown are the major bioinformatical steps to create an input data set (A), steps for potential candidate gene identification (B), and the computational steps to create the candidate SCO data sets as well as their three annotation comparisons (C).

We used RNA sequencing data from the electric organ (EO) of three *Campylomormyrus* species (*C*. *compressirostris*, *C*. *tshokwe* and *C*. *tamandua)* and the closely related species *G*. *petersii* to assemble tissue-specific transcriptomes (Fig 5A). We downloaded the Illumina raw reads from the Sequence Read Archive (SRA; http://www.ncbi.nlm.nih.gov/sra) with the accession number SRP050174 [40]. The processing of raw reads (quality filter, adapter trimming, etc.) was achieved as described in [40]. Filtered paired-end reads of the four species were assembled de novo into separate transcriptomes using Trinity v. 2.2.0 with default parameters [76]. The four tissue-specific assemblies were tested for transcriptome completeness using BUSCO v3 [77]. For this purpose, transcriptomes were compared to the core gene set of Actinopterygii (state: 2018). The assemblies had been analyzed with the TransDecoder 3.0.1 pipeline to obtain the longest open reading frame for the transcripts [78]. The four transcriptomes served as input for the subsequent orthology analysis for which Orthofinder 1.1.10 was used (Fig 5) [79]. The Orthofinder tool is based on an all-versus-all blast of amino acid sequences, followed by a first sequence clustering taking into account the normalized bit score of the blast results. Afterwards, orthologous genes are selected and a final clustering by the Markov Cluster algorithm results in discrete orthogroups. Orthofinder distinguished between orthogroups with multiple sequences per species and Single Copy Orthogroups (SCO; one sequence per species). Further analyses were applied only to the SCOs to ensure analytical comparison among orthologous genes. The four nucleotide sequences of each SCO had been aligned codon-wise using PRANK v. 140110 (default parameters) [80] and trimmed to equal length by a customer bash script. To discard remaining paralogs, YASS 1.15 [81] was used. It compared all sequences pair-wisely and outputs the similarity for each pair in percentage. Single copy orthogroups with a similarity value below 90% were discarded from our data set. This procedure ensured that our retained genes are either clearly distinguishable from an ancient paralog (i.e., identified as separate SCOs) or do not have a paralog, at least not expressed in the electric organ. Furthermore, sequences shorter than 200 bp were removed. Finally, a randomly chosen subset of SCOs was checked manually for correct filter criteria confirming the performance of the bioinformatical scripts and tools. The resulting data set of SCOs served as basis for our subsequent analyses (Fig 5).

### Approach I: Identification of candidate genes potentially related to EOD elongation in *C*. *tshokwe*

First, the ratio of non-synonymous (dN) to synonymous (dS) nucleotide substitution rates was calculated for all SCOs using the codeml package implemented in PAML v. 4.9 [82]. Therefore, the sequence alignments of each SCOs were loaded to codeml separately and the site model M0 was chosen to compute the respective omega value (ω = dN/dS). The ω value is an indicator of selective pressures on genes. A ratio significantly greater than 1 indicates positive selection. A ratio of 1 indicates neutral evolution at the protein level. A ratio less than 1 indicates selection to conserve the protein sequence (i.e., purifying selection). By using Geneious R 8.1.9, SCOs with ω > 1 were screened manually for non-synonymous species-specific SNPs occurring only in the sequence of *C*. *tshokwe* (elongated EOD), when compared to the species with short EOD (*C*. *compressirostris*, *C*. *tamandua* and *G*. *petersii;* Fig 5B). As *C*. *tshokwe* and *C*. *compressirostris* are closely related, the direct comparison of these two species allowed us to focus on genes which experienced genetic changes since they diverged from their last common ancestor. Single Copy Orthogroups with ω > 1 and non-synonymous, species-specific SNPs in *C*. *tshokwe* were considered as putative candidate genes for an elongated EOD (Fig 5B). Nucleotide sequences of these SCOs were blasted against the current nucleotide database of NCBI (nt database) using the BLASTn algorithm [83]. We also blasted sequences against the protein database of the Zebrafish Information Center (ZFIN) and the UniProtKB/Swiss-Prot database using the BLASTx algorithm. Putative candidate genes were assigned to GO terms using Blast2GO version 5.2.4 (java version) [84]. Gene ontology terms represent a controlled vocabulary of gene attributes which are organized hierarchically with three top categories: Biological Process; Molecular Function; Cellular Component. In order to identify biological pathways in which the candidate genes occur, we uploaded the nucleotide sequences to the Kyoto Encyclopedia of Genes and Genomes Automatic Annotation Server (KAAS) [85] and blasted them against the gene data base of all available fish species as well as the human (*Homo sapiens*) and mouse (*Mus musculus*) data bases. Each run was performed with a bi-directional best hit algorithm (BBH). The Kyoto Encyclopedia of Genes and Genomes (KEGG) is a database of molecular functions which stores orthologs of experimentally characterized genes and proteins which are included in different biological processes (KEGG pathways). Each unit of these pathways is defined by a KO number. The KO numbers can be pooled by pathways which in turn can be grouped to level B categories. Several level B categories form a level A category. KEGG distinguishes six level A categories: 'Metabolism', 'Genetic Information Processing', 'Environmental Information Processing', 'Cellular Processes', 'Organismal Systems', and 'Human Diseases'. We applied a multivariate analysis of protein polymorphisms (MAPP) for the same gene set to detect impaired amino acids in the sequence. MAPP calculates a score to predict the impact of amino acid substitutions on protein function and structure. This impact score considers the properties of the amino acids and the phylogenetic relationship in the appropriated gene (gene tree) [86].

### Approach II: Annotation comparisons of orthogroups between *C*. *compressirostri*s and *C*. *tshokwe*

The second approach aimed at identifying cellular and molecular mechanisms which play a role in the differentiation of EOD duration. Therefore, all SCO alignments were converted into a VCF file format using the tool SNP-sites to call SNPs from multiple sequence alignments [87]. Singe copy orthogroups with non-synonymous species-specific SNPs in *C*. *tshokwe* and *C*. *compressirostris* were isolated separately, creating two candidate SCO data sets. Subsequently, ω values for each remaining SCO were determined with the Ka/Ks Calculator 2.0 using the model selection according to the AICs (MS) [88]. Its underlying calculation deviates from the M0 site model (codeml), as it relies on a pairwise calculation across all input sequences and outputs a ω value for each possible combination. Only SCO with ω > 1 for the pairing of *C*. *tshokwe* and *C*. *compressirostris* were retained in the candidate SCO data sets (Fig 5C). As we wanted to look for over-/underrepresentation of candidate SCO-sequences in certain biological processes, the candidate SCO data sets of both species were analyzed separately by a GO term annotation using Blast2GO version 5.2.4. Furthermore, KEGG orthology annotations were performed for both candidate SCO data sets as well as for both transcriptomes, considering for any gene only the transcript with the longest open reading frame (Fig 5C). We uploaded each data set separately to KAAS and used the same parameters and databases as in approach I. The GO term and KEGG pathway annotations were used for four annotation comparisons (Fig 5C).

The species-specific candidate SCO data sets were sorted by GO terms and the number of candidate SCO-sequences in each GO term was determined. Their proportion (in their respective candidate SCO data set) was compared among the two species. To account for different absolute sequence numbers underlying this comparison, we determined 95% confidence intervals of proportions at an equal ratio (50:50) for different total numbers of sequences (2 ≤ n ≤ 200) (S5 Table). Upper and lower confidence intervals were plotted using R version 3.4.4 (R Development Core Team, 2008). To identify the GO terms with a significant difference among *C*. *tshokwe* and *C*. *compressirostris*, we calculated the proportional ratio of candidate SCO-sequences between both species for each GO term and plotted them against the confidence interval, rendering GO terms outside the confidence interval significant. For the second analysis (KEGG annotation comparison), total numbers of candidate SCO-sequences in each KEGG level A category were compared among the two species and significant differences were tested with the Fisher Exact Test (α = 5%). In addition, for each KEGG level A category, it was tested with the Chi^2^ test (α = 5%) whether candidate SCO-sequences were assigned disproportionally to the level B categories (next lower hierarchical KEGG level) among the two species. For a closer look on the KEGG annotations, we grouped the pathways by KEGG level B categories and counted the candidate SCO-sequences in each KEGG level B category for both data sets. Here, KEGG categories were tested for significance among *C*. *tshokwe* and *C*. *compressirostris* analogous to the GO terms, i.e., identification of outliers to the 95% confidence interval of proportions. Data were visualized using R version 3.4.4. We further evaluated whether certain GO terms or KEGG Level A categories are enriched among the candidate SCO-sequences, compared to the respective transcriptome (Fig 5C). The enrichment analysis of Blast2GO (Fisher’s Exact test; FDR = 0.05) was used to identify over- or underrepresented GO terms in the candidate SCO data set. We applied this analysis to both species separately. To reveal KEGG level A categories with significant differences between the candidate SCO and transcriptome data set, the sequence number which matched each of the 6 KEGG level A categories were determined in each single data set separately (2x candidate SCO data sets and 2x transcriptome data sets). Their proportions (in their respective data set) were calculated and the respective 95% confidence intervals were determined using the online tool of the Allto Market Research web site [89]. The data were illustrated as bar chart with Microsoft Excel 2010.

## Supporting information

S1 Fig GO term annotations regarding the 27 candidate genes of *C*. *tshokwe*. Pie charts depict the proportion of sequences annotated to the main GO categories Biological Process (A), Cellular Component (B) and Molecular Function (C).
(TIF)

S1 Table KEGG orthology annotation matching 7 candidate genes of *C*. *tshokwe*. For each gene, KO number and KEGG level A and B categories as well as pathways are listed.
(XLSX)

S2 Table List of GO terms occurring in the candidate SCO data sets of *C*. *tshokwe* and *C*. *compressirostris*. 141 GO term names and IDs, corresponding absolute number of candidate SCO-sequences and relative proportions of both species and for each GO term are given.
(XLSX)

S3 Table List of inferred KEGG pathways of both candidate SCO data sets for *C*. *tshokwe* and *C*. *compressirostris*. Raw data are separated by sheets. Shared and species-specific KEGG pathways, their level B categories and corresponding number of candidate SCO-sequences for both species are given.
(XLSX)

S4 Table Confidence interval of proportion among KEGG results of the candidate SCO data sets and the respective transcriptome. Sequence counts [%] matching the 6 KEGG level A categories and their respective confidence intervals are listed at a 5% level are listed. Data are available for the candidate SCO data sets and the transcriptomes (LORF) in *C*. *tshokwe* and *C*. *compressirostris*.
(XLSX)

S5 Table Upper and lower confidence intervals of proportion for total numbers (Approach II). (XLSX)
