# Supplementary figures and images for: Transcriptome-wide single nucleotide polymorphisms related to electric organ discharge differentiation among African weakly electric fish species
Source: PLoS One. 2020 Oct 27;15(10):e0240812. doi: 10.1371/journal.pone.0240812 (PMC7591079; doi:10.1371/journal.pone.0240812)

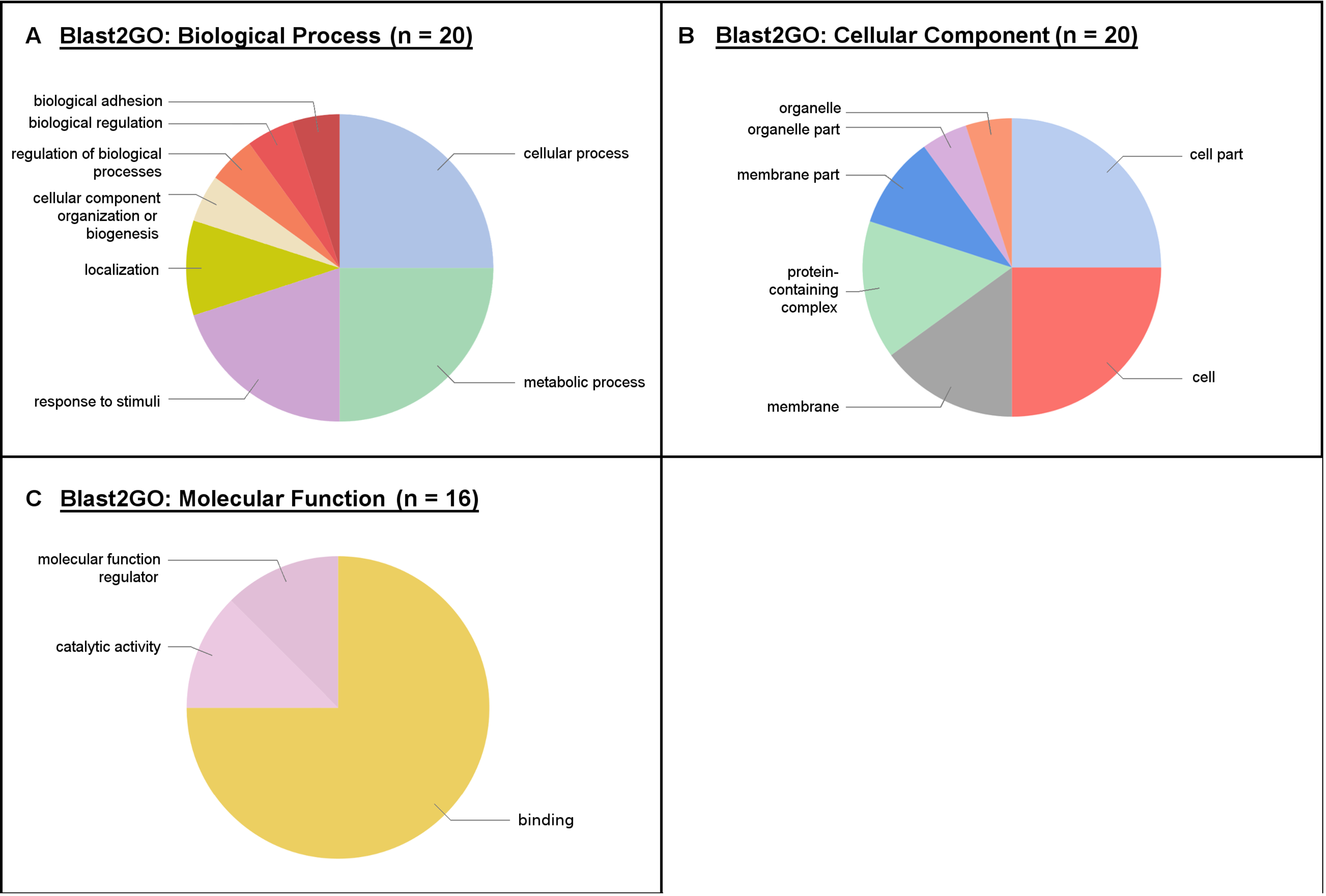

Supplement: S1 Fig — Pie charts depict the proportion of sequences annotated to the main GO categories Biological Process (A), Cellular Component (B) and Molecular Function (C). (TIF) [file pone.0240812.s001.tif]
